# Cation Effects on Interfacial Water Structure and Hydrogen Peroxide Reduction on Pt(111)

**DOI:** 10.1021/acsmeasuresciau.1c00004

**Published:** 2021-07-07

**Authors:** Valentín Briega-Martos, Francisco J. Sarabia, Víctor Climent, Enrique Herrero, Juan M. Feliu

**Affiliations:** Instituto de Electroquímica, Universidad de Alicante, Apdo. 99, E-03080 Alicante, Spain

**Keywords:** platinum single crystals, alkali metal cations, electrocatalysis, electrical double layer, laser-induced temperature jump, electrochemical interface

## Abstract

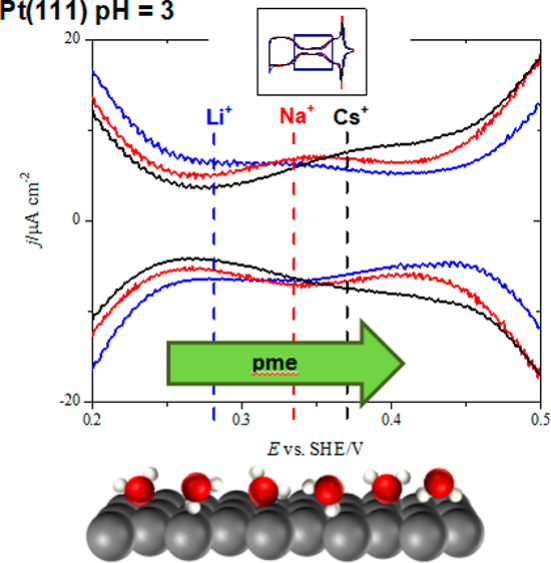

The interface between the Pt(111) surface and several MeF/HClO_4_ (Me^+^ = Li^+^, Na^+^, or Cs^+^) aqueous electrolytes is investigated by means of cyclic voltammetry and laser-induced temperature jump experiments. Results point out that the effect of the electrolyte on the interfacial water structure is different depending on the nature of the metal alkali cation, with the values of the potential of maximum entropy (pme) following the order pme (Li^+^) < pme (Na^+^) < pme (Cs^+^). In addition, the hydrogen peroxide reduction reaction is studied under these conditions. This reaction is inhibited at low potentials as a consequence of the build up of negative charges on the electrode surface. The potential where this inhibition takes place (*E*_inhibition_) follows the same trend as the pme. These results evidence that the activity of an electrocatalytic reaction can depend to great extent on the structure of the interfacial water adlayer and that the latter can be modulated by the nature of the alkali metal cation.

## 1 Introduction

The properties of the metal|aqueous solution interphase have a key influence on the rate of electrocatalytic reactions.^1^ Among them, the charge separation in this narrow region is of paramount importance since it induces a strong electric field that affects the molecules and ions near the electrode surface.^2,3^ The surface of the metal is covered with a layer of water dipoles polarized by the electric field that influences the electrocatalytic activity of the electrode by strongly affecting the movement and interactions of reactants and products across the electrified interphase. The electrocatalysis will then be determined by both the interfacial water structure and its interaction with the reactive species as well as with the metal electrode surface.

In order to perform fundamental studies about the effects of the surface charge and the interfacial water structure on electrocatalysis, the use of Pt single-crystal electrodes with well-defined surfaces is mandatory since the surface structure strongly affects these parameters and their influence on an electrocatalytic reaction. The relationship between surface charge and electrode potential can be established by knowing, in addition to the differential capacity, the potential of zero charge (pzc) of the electrode surface.^4^ However, in the case of Pt, where the specific adsorption of species results in a transfer of charge through the electrochemical interphase, the determination of the pzc becomes more challenging. For this situation, two types of interfacial charges are defined: free charge (σ), which is the true charge density on the metal surface compensated by the ions in the electrolyte, and total charge (*q*), which includes the free charge and also the charge involved in adsorption processes.^4,5^ The electric field at the interphase and, therefore, the dipole orientation of the interfacial water molecules are mainly governed by the free charge. In correspondence with the two different charge definitions, two different values of pzc should be considered: the potential of zero free charge (pzfc) and the potential of zero total charge (pztc).^5^ The pztc is the one accessible from electrochemical measurements, i.e., by CO displacement experiments, and can be determined using a pure thermodynamic approach, whereas the pzfc can be estimated for Pt(111) from the pztc within a model after some non-thermodynamic assumptions. This determination involves an extrapolation of the differential capacity into the regions of hydrogen or hydroxyl adsorption, and therefore, the obtained value can more accurately be called the potential of zero extrapolated charge (pzec).^5,6^

The pzfc can also be estimated from additional indirect measurements. On the one hand, peroxodisulfate (PDS) has been proposed as a local probe for the estimation of the pzfc on Pt surfaces. Given that PDS is an anionic species, the drop in the current of PDS reduction corresponds to the point where the surface charge changes from positive to negative values.^7,8^ On the other hand, the pzfc can be inferred employing laser-induced temperature jump (LITJ) experiments.^9,10^ This methodology employs high-energy laser pulses of nanosecond duration for increasing the temperature at the interface. The transient response to this perturbation under coulostatic conditions provides information about the net orientation of water molecules at the interphase. The potential where the transient is flat corresponds to the situation in which the water molecules are totally disordered, and therefore, this potential is called the potential of maximum entropy (pme) of double-layer formation. The pzfc can be approximated to the pme if the electrostatic forces dominate the orientation of water molecules, as has been demonstrated to be the case in several studies.^11−16^ In this case, the situation of zero charge would lead to a totally disordered water adlayer. Therefore, the LITJ methodology can provide valuable information about the surface electric field and the orientation of water dipoles at the interphase.^9,10,17,18^

The study of the effect of the pH on the surface charge and its influence on the interfacial water orientation is of great interest. Such a study was possible on Pt(111) within the pH range between 1 and 6 using NaF/HClO_4_ mixtures as buffer solutions as they avoid the specific adsorption of anions. Using LITJ measurements in these conditions, it was demonstrated that the pme is nearly constant in the SHE scale in the whole studied pH range, with a value of 0.320 ± 0.030 V.^19^ These results agree with the obtained pzec values from CO displacement experiments^6^ and with the estimated pzfc values from PDS reduction measurements in the same buffer conditions.^8,20^ These studies allowed establishing relationships between surface charge and interfacial water structure, as well as the activity of some important electrocatalytic reactions, including the oxygen reduction reaction (ORR) and the hydrogen peroxide reduction reaction (HPRR),^21−23^ hydrogen evolution and oxidation reactions (HER/HOR),^24,25^ and methanol oxidation reaction (MOR).^26^

The above-mentioned studies with NaF/HClO_4_ mixtures were carried out in the presence of only Na^+^ or both Na^+^ and K^+^ (using KClO_4_ for completing the ionic strength), but a systematic study on how the nature of different cations affects these measurements has not been performed up to now. The first evidence of the influence of the nature of the cations on the structure of the electrochemical interface for Pt single-crystal electrodes involves the investigation of cation effects in phosphate and sulfuric acid solutions, and in light of these results, the authors proposed the existence of specific cation–anion interactions.^27−29^ Density functional theory (DFT) calculations in combination with cyclic voltammetry experiments with well-defined Pt surfaces were used to study the effects of pH and an alkali cation on the cyclic voltammograms of Pt electrodes.^30−33^ Bandarenka et al. studied the influence of the nature of the alkali metal cations on the electric double-layer capacitance for different well-defined Pt, Au, and Cu surfaces, and the results pointed out that the differential EDL capacitance measured close to their pzc increased linearly following the order Li^+^ < Na^+^ < K^+^ < Rb^+^ < Cs^+^.^34,35^ Marković et al. investigated the specific cationic effects on the ORR, HOR, and MOR in alkaline media,^36^ and after this study, several works investigating the effects of the nature and concentration of cations on electrocatalytic reactions of interest in different metals have been reported.^37−51^

It should be emphasized that interfacial water can also play an important role as a reagent in the forms of H_2_O or H_3_O^+^. It may happen that only the water near the surface with a given orientation is reactive, and not all of the water at the interphase is available for a reaction in a very short time. Therefore, only a fraction of water would be reactive. In addition, the charge separation at the electrical double layer can determine the orientation of the relevant water molecules. Apart from the electrode charge, which is the most important contribution, cations in the solution also create an electric field that can influence the orientation of water, increasing or reducing its availability as a reagent at the interphase. The influence of the presence of ions on the structure of water has also been extensively studied in the literature.^52−55^

In the present work, the effect of the nature of the cation present in the solution on the interfacial water structure for Pt(111) is studied by means of LITJ measurements in MeF/HClO_4_ solutions, with pH values ranging from 2 to 4.5. The possible differences in reactivity for the HPRR, especially on the current inhibition at low potentials, induced by changes in the interfacial water molecules as a consequence of the different present cations are also investigated.

## 2 Experimental Section

### 2.1 Cyclic Voltammetry and HPRR Measurements

Cyclic voltammetry experiments and HPRR measurements were carried out in a two-compartment electrochemical glass cell with three electrodes according to the general procedure detailed in ref (56). The Pt(111) working electrode was prepared from a small Pt bead of ∼2 mm in diameter following the method described by Clavilier et al.^57^ The electrode was flame annealed in a propane flame, cooled in Ar/H_2_ (3:1) reducing atmosphere, and protected with an ultrapure water drop saturated with these gases before its transference to the electrochemical cell. The counter electrode was a Pt coiled wire cleaned by flame annealing and quenched with ultrapure water. The reference electrode was a Ag/AgCl, KCl (saturated) electrode.^21^ Potential values have been transformed to RHE or SHE when required. For doing this and for determining with precision the pH of the working solutions, before every series of measurements, a NaH_2_PO_4_/Na_2_HPO_4_ buffer solution with pH ∼7 was prepared, and its pH was exactly measured with a pH meter. Since the calibration standards for the pH meter have pH values of 4, 7, and 9, the pH of the prepared NaH_2_PO_4_/Na_2_HPO_4_ buffer solution lies right in the middle of the calibration curve, and therefore the measurements are very reliable. After this, the employed Ag/AgCl reference electrode is measured against a Pt wire in the same NaH_2_PO_4_/Na_2_HPO_4_ buffer solution saturated with H_2_. In this way, since the pH of the solution is well-known, the standard potential of the reference electrode can be precisely determined. Once this is done, the potential difference between the reference electrode and Pt wire is measured in the corresponding working solution subject to study saturated with H_2_. Since the standard potential of the reference electrode is known, the pH of the working solution can be electrochemically measured with high precision, and with these data, the conversions to SHE and RHE can be done. This procedure was also explained in ref (21).

Working solutions were prepared using concentrated HClO_4_ (Merck, for analysis), LiF and NaF (Merck, Suprapur, 99.99%), CsF (Alfa Aesar, Puratronic 99.99% (metal basis)), and ≥30% H_2_O_2_ solution (Fluka, TraceSELECT Ultra, for trace analysis). Ar was employed for the deoxygenation of the working electrolyte. Ultrapure water (Elga PureLab Ultra, 18.2 MΩ·cm) was used for glassware cleaning and the preparation of the solutions.

An EG&G PARC signal generator and an eDAQ EA161 potentiostat with an eDAQ e-corder ED401 recording system were used for the electrochemical measurements. Experiments in hydrodynamic conditions were carried out in the HMRDE configuration using an EDI101 rotating electrode. The rotating rate was controlled with a CTV 101 radiometer. For further details about the kinetic equations and the experimental arrangement for this particular configuration, please refer to refs (58) and (59). All experiments were performed at room temperature.

### 2.2 Laser-Induced Jump Temperature Method

The laser-induced jump temperature method provides information related to the sign and magnitude of the interfacial electric field. The details of this method are given elsewhere.^9,15^ The experiments were carried out in an electrochemical cell with a fourth electrode in addition to those typically used in a conventional electrochemical system (counter, reference, and working electrode). The fourth electrode is a Pt wire polarized at the same potential as the working electrode. In this technique, a thermal perturbation is produced at the surface of the working electrode using a pulsed laser. The working electrode is disconnected from the potentiostat 200 μs before triggering the laser, leaving the system at open-circuit potential. After successive laser pulses, the working electrode is connected again at the previous potential value. In this way, the change in the working electrode potential due to the increase of temperature is recorded under coulostatic conditions (constant charge) by measuring the potential difference between the working electrode and the fourth additional electrode.

The employed light source was a Brilliant Q-switched Nd:YAG laser (Quantel) that provides laser pulses of 5 ns with a frequency of 532 nm. This source was synchronized with a Tektronix model TDS 3054B oscilloscope and a potentiostat–galvanostat. The oscilloscope provides an average measurement after recording 250 potential transients at each desired potential. The energy of the laser was 16 mJ/cm^2^, and it was regulated by combining the effect of an attenuator from Newport Corporation (model M-935-10) and the Q-switched time. The laser energy was measured by collecting the beam in a pyroelectric sensor head (model LM-P10i). On the other side, a system of convergent and divergent lenses was used to control the diameter of the laser (4 mm).

## 3 Results and Discussion

**Figure 1:**
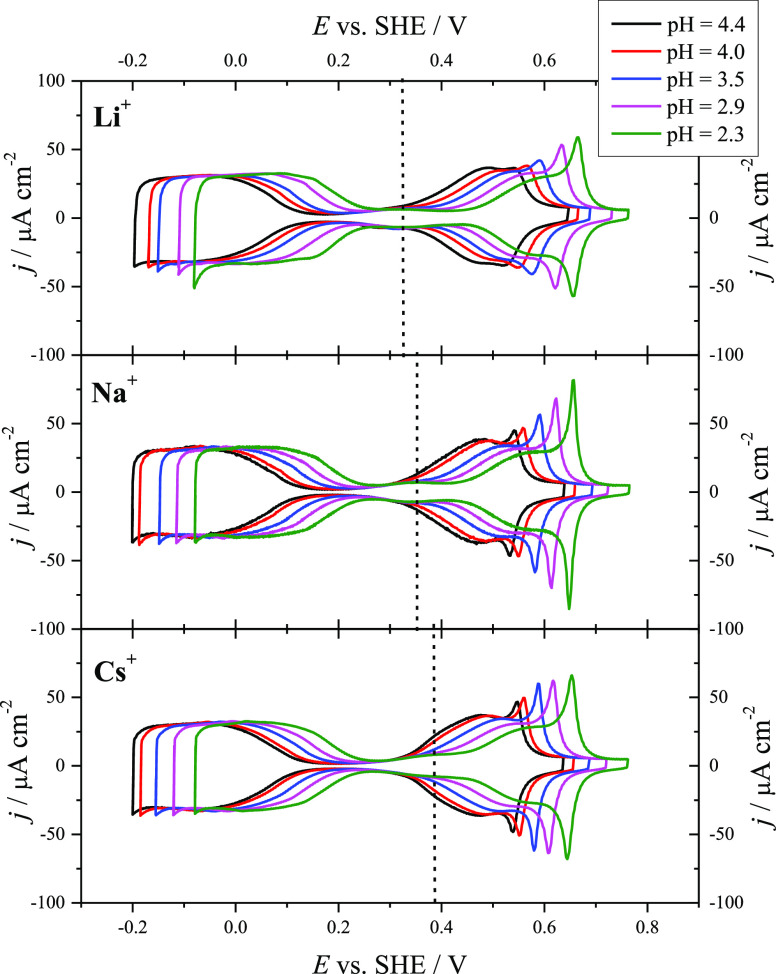
Cyclic voltammograms for Pt(111) in Ar-saturated MeF/HClO_4_ mixtures (Me^+^ = Li^+^ (top), Na^+^ (center), Cs^+^ (bottom)) for pH 4.4 (black), 4.0 (red), 3.5 (blue), 2.9 (magenta), and 2.3 (green). Scan rate: 50 mV s^–1^. The vertical dashed lines indicate the maximum of the reversible broad peak in the double-layer region for each metal alkali cation.

Figure 1 shows the cyclic voltammetry results for Pt(111) in buffer solutions with 0.1 M MeF in the case of NaF and CsF or with 0.04 M Li in the pH range from 4.4 to 2.3. The lower concentration in the case of LiF is due to the limited solubility of this salt. It should be emphasized that cyclic voltammetric profiles in both NaF and CsF in 0.1 and 0.04 M are nearly identical, suggesting that there will not be important differences in the behavior between these concentration values. A reversible broad peak can be observed in the double-layer region between the hydrogen and OH adsorption/desorption regions. This feature was already observed for HClO_4_ + KClO_4_ solutions with pH values up to 3,^9,10,60^ and it was attributed to the reorientation of the interfacial water molecules.^9,60−62^ In fact, the maximum of this peak is close to the pme for Pt(111) as determined by LITJ experiments in the mentioned conditions.^10^ Cyclic voltammetry experiments with NaF/HClO_4_/KClO_4_ mixtures also showed this feature for Pt(111) at neutral pH values up to 5.^6,19,21^ In all of these previous works, the position of the reversible broad peak is essentially the same despite the different concentrations and nature (Na^+^ or K^+^) of the alkali metal cations, being centered at ∼0.36 V vs SHE. It only has been noticed that in the absence or very low concentrations (below 10^–3^ M) of alkali metal cations this feature disappears, and under these conditions, a Gouy–Chapman capacitance minimum can be observed at pH 3.^63^ Double-layer models including attracting ion–surface interactions site effect are under the scope to tune Gouy–Chapman capacitance within the framework of the Stern model of the interface.^64^ However, a dependence of the peak potential of the reversible broad peak with the nature of the alkali metal cation is pointed out for the first time in Figure 1. It can be observed that the maximum of the broad peak is shifted to more positive potentials in the order Li^+^ < Na^+^ < Cs^+^. Since this feature is related to the water reorientation at the interface, these results clearly point out an influence of the nature of the alkali metal cation on the interfacial water structure. Additional effects can be observed in the voltammetric profiles at constant pH values. As shown in Figure S1, the hydrogen adsorption region is not affected by the main cation in solution. However, some changes can be observed in the OH adsorption region. For Li^+^, the onset of OH adsorption is displaced to higher potential values, and the peak at ∼0.8 V is less sharp. These changes are in agreement with the proposed effect of the kosmotrope and chaotrope ions in this region.^65^ For the considered cations, the order starting from kosmotropes to chaotropes is Li^+^ < Na^+^ < Cs^+^. The effect of kosmotrope ions is the diminution of the peak at 0.8 V, as observed for Li^+^.

In order to confirm the effect of the nature of the alkali metal cation on the interfacial water molecules, LITJ experiments have been performed in the same electrolytes used for the cyclic voltammetry experiment in Figure 1, as these measurements constitute a direct investigation of the interfacial water properties. Figures S2–S4 reflect the Δ*E* vs *t* laser transients recorded for Pt(111) at several potentials for each one of the cations employed in this work at different pH values.

In order to get a more accurate and quantitative analysis of the interfacial electric field, the calculation of the values of the thermal coefficient, , is required. In the case of a fast response of the double layer to the thermal perturbation once the laser reaches the electrode surface, the change of potential (Δ*E*) at the interface is proportional to the change of temperature (Δ*T*), and the proportionality constant is given by the thermal coefficient (eq 1):^66^
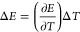
1

Δ*T* depends on the duration of the laser pulse, *t*_0_, which in this work is 5 ns, the relaxation time after the pulse, *t*, and the initial change of temperature, Δ*T*_0_, according to eq 2:
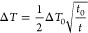
2Δ*T*_0_ can be theoretically obtained through a model using the following eq 3:
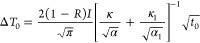
3where *I* is the laser intensity, *R* is the reflectivity of the surface and α, κ and α_1_, κ_1_ are the thermal diffusivity and thermal conductivity of the metal and the aqueous solution, respectively (α = κ/ρ*c*, where ρ is the density and *c* is the heat capacity). All these values are given in Table S1. The obtained value for Δ*T*_0_ in the present conditions is 58 (in temperature units).

Finally, the equation that relates Δ*E* with *t* (eq 4) can be expressed as follows by the combination of eq 1 and eq 2:
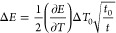
4

**Figure 2:**
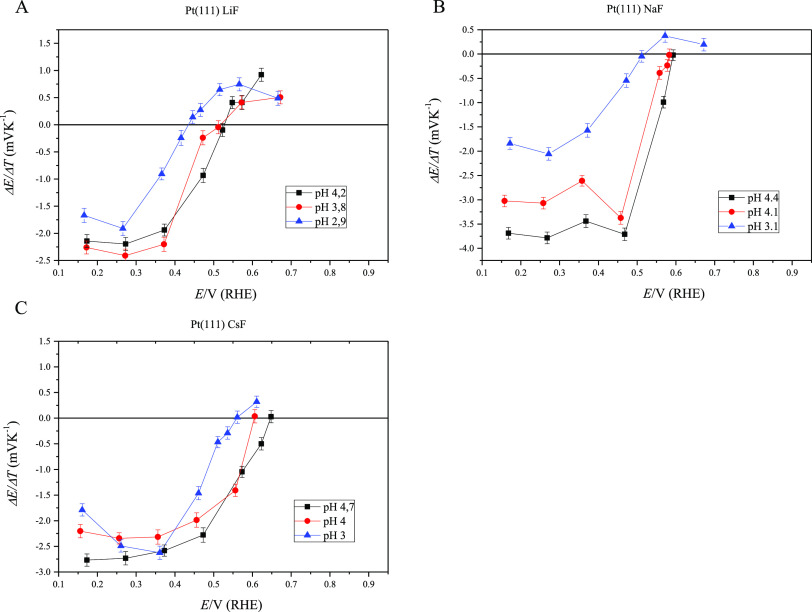
Thermal coefficients for the Pt(111) electrode for each one of the cations (Li^+^ (A), Na^+^ (B) and Cs^+^ (C)) in solution at their respective pH values. The plotted thermal coefficients have been obtained from the laser transients reflecting a single contribution since the linearization Δ*E* vs 1/√*t* only is possible for a single fast process. The negative contribution appearing at potentials above the pme is due to the effect of the fluoride anion as it has been previously reported.^19^

According to eq 4, for the double-layer formation can be obtained from the resulting slopes of the Δ*E* vs *t*^–1/2^ plots, which are shown in Figure S5. Figure 2 shows the values for the different applied potentials at each studied pH for the corresponding cation. In addition, the thermodiffusion effect as a consequence of the difference of temperature between the reference and the working electrode has been taken into account. However, in our solution conditions, these values of thermodiffusion coefficients have a negligible effect on the thermal coefficients and the pme values. These values were calculated, such as it has been explained elsewhere,^66^ from the mobility and the Eastman entropies of transport of the ions for the different concentrations of the ions in solution for each experiment.^67,68^ The pme values have been obtained from the potential in which the plot of vs *E* reaches the zero value. On the other side, it is worth highlighting that the thermal coefficients shown in Figure 2 correspond to the laser transients which reflect a monotonic behavior, or in other words, to the transient without bipolarity due to presence of processes with different time constants.

**Figure 3:**
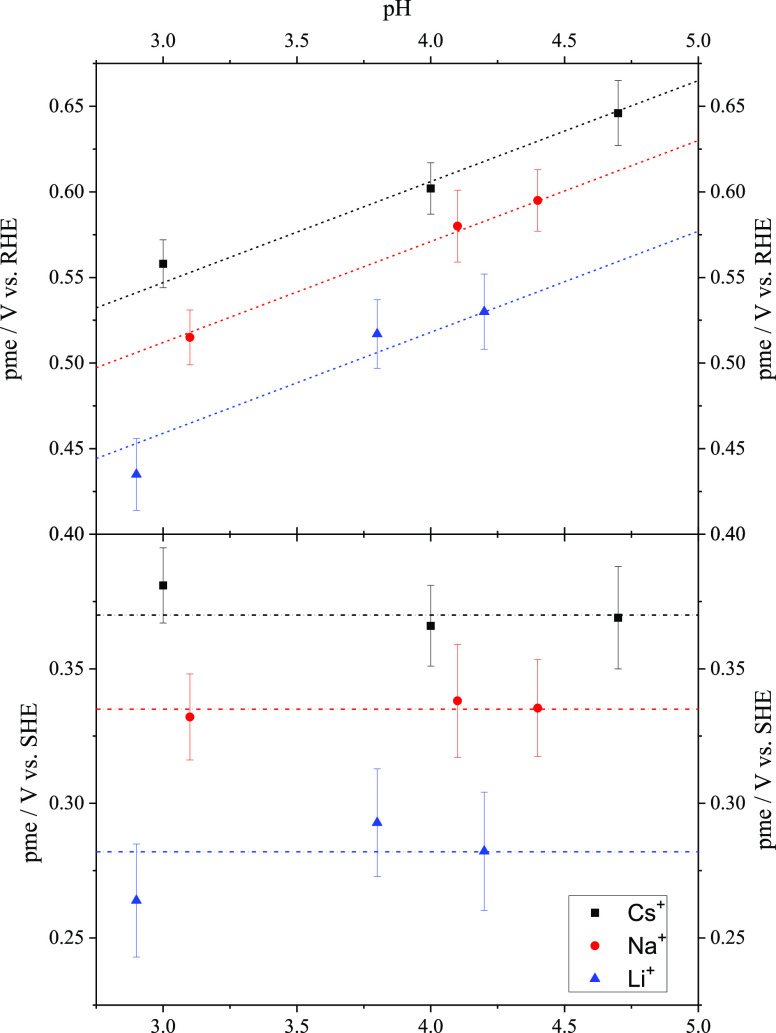
Plot of the pme values calculated for Pt(111) in Ar-saturated MeF/HClO_4_ mixtures (Me^+^ = Li^+^ (blue triangles), Na^+^ (red circles), Cs^+^ (black squares)) for different pH values at both RHE (top) and SHE (bottom). The dashed and dashed dotted lines correspond to slopes of 59 and 0 mV/decade, respectively, assuming the pme is 370 mV vs SHE for Cs^+^, 335 mV vs SHE for Na^+^, and 282 mV vs SHE for Li^+^.

The corresponding extracted pme values from the thermal coefficients are presented in Figure 3. The same trend as that for the reversible broad peak is observed: the pme values are the same in the SHE scale for all pH values (or they increase 59 mV per pH unit in the RHE scale) for each alkali metal cation, and they follow the order pme (Cs^+^) > pme (Na^+^) > pme (Li^+^). The data for the NaF-containing electrolyte agree with the previous results in these pH values. Measured values for the pme are always ∼20 mV more negative than the potential for the maximum of the broad reversible feature, which was also observed in the previous works with NaF/HClO_4_ mixtures.^19,69^ In order to explain the shift of the maximum of the broad reversible feature with respect to the pme is important to keep in mind the relationship between the capacitance of the EDL and the structure of the interfacial water network. It has been previously proposed that the changes in the capacitance of the inner layer of the interface are due to an electrostatic phenomenon of deformation of the structure of the dipoles at the interface, known as electrostriction, leading to a change of interfacial water structure.^70,71^ In addition, this requires a preferential orientation of the water adlayer with the oxygen atom pointing toward the electrode surface at the pzc. In this regard, studies carried out on mercury and gold surfaces reported that water molecules present a natural orientation with the oxygen pointing away from the metal at the pzfc because of specific interactions between the oxygen atoms and the electrode surface.^12−14^ In this way, it is reasonable to assume the maximum of the broad feature appearing in the double-layer region is closely related to the pzfc. Then, it would be necessary to apply a negative charge, i.e., a lower potential on the surface to diminish the negative dipolar contribution associated with the oxygen atoms in order to reach the pme. This is the first time that the influence of the nature of the metal alkali cation on the structure of the interfacial water molecules has been evidenced directly by LITJ measurements. Previous publications focused on the effect of the surface structure and pH, and only some effects of the fluoride anions were addressed in the previous work by Sebastián et al.^19^

**Figure 4:**
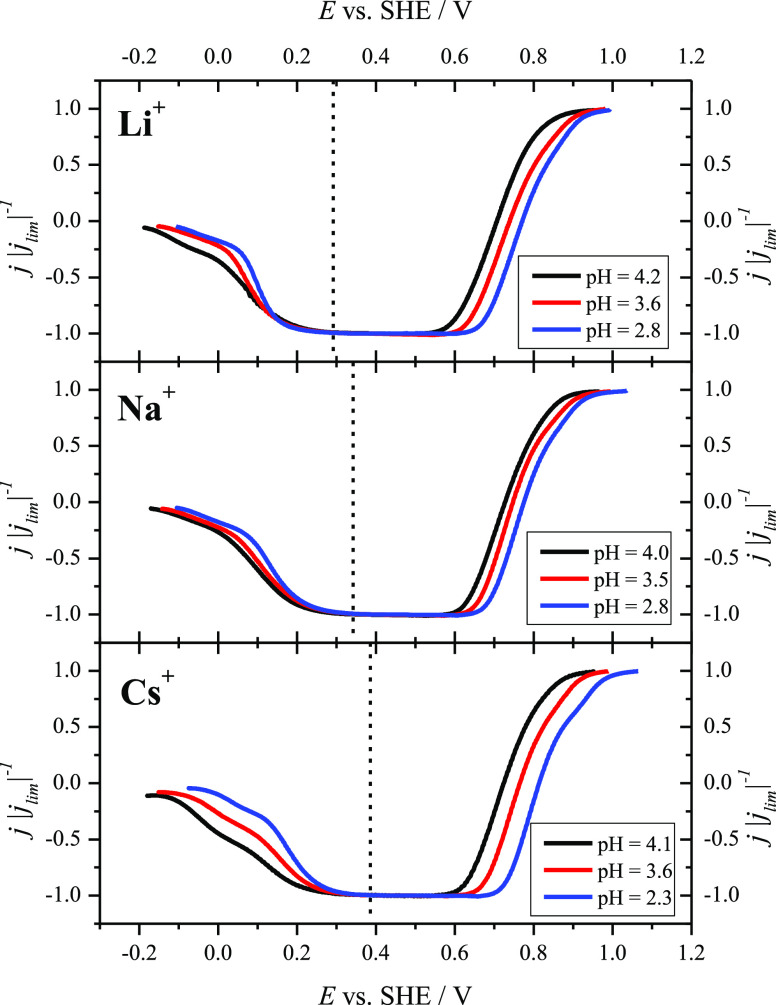
Normalized polarization curves for the HPRR and HPOR on Pt(111) in Ar-saturated MeF/HClO_4_ mixtures (Me^+^ = Li^+^ (top), Na^+^ (center), Cs^+^ (bottom)) with 1.7 mM H_2_O_2_ for different pH values ranging from 2.3 and 4.1. Rotation rate: 2500 rpm; scan rate: 50 mV s^–1^. The vertical dashed lines indicate *E*_inhibition_ for each metal alkali cation.

The HPRR in the present conditions has been studied using the HMRDE configuration at 2500 rpm, and the results are shown in Figure 4. A previous study using NaF/HClO_4_/KClO_4_ mixtures of different pH values from 2 to 5.6 showed that the potential at which the inhibition of the HPRR takes place on Pt(111) (*E*_inhibition_) is invariant in the SHE scale.^72^ This potential nearly coincides with the position of the peak for the reversible broad feature, which is in turn intimately related to the pme for this surface. Based on these results, a relationship is evidenced between the inhibition of the HPRR at low potentials and the changes in the interfacial water structure created by the electric field. Similar correspondence was also observed for the local pme for steps and terraces on Pt stepped surfaces (near the local pme for steps, there is a reactivation of the reduction current).^23^ Therefore, variations in *E*_inhibition_ depending on the metal alkali cation present in the solution can be directly related to changes in the interfacial water structure induced by the different nature of the cation. In Figure 4, exactly the same correspondence can be observed between the potential for the maximum of the reversible broad feature (Figure 1) and *E*_inhibition_ for the HPRR for each of the studied metal alkali cations. Therefore, *E*_inhibition_ for the different metal alkali cations follows the order *E*_inhibition_ (Cs^+^) > *E*_inhibition_ (Na^+^) > *E*_inhibition_ (Li^+^), which is the same as that observed for the peak potential for the reversible broad feature and the pme. The main conclusion that can be drawn from these results is that the metal alkali cations affect differently the interfacial water structure depending on their nature, and the interfacial water structure, in turn, influences the inhibition of the HPRR. In this way, the present results represent direct evidence of the influence of the nature of metal alkali cations on the activity of an electrocatalytic reaction through changes in the interfacial water structure. The effect of the cation is not only restricted to the region where the inhibition takes place. Also, the potential for which the current is zero, that is, the potential at which the rate for the HPOR and HPRR are the same, follows *E* (Cs^+^) > *E* (Na^+^) > *E* (Li^+^) (Figures S6 and S7), which suggests that the surface charge also plays a role in this region, in the same way that the OH adsorption has been affected. However, the detailed analysis of the effect of the metal alkali cation on the kinetics of the HPRR and HPOR is more complicated. On the one hand, the study of interfacial water structure at high potentials is more difficult because the faradaic contribution from OH adsorption affects the measurements with the LITJ technique. On the other hand, as mentioned before, the surface oxides play a role in the kinetics of these reactions, so it will be difficult to analyze the effect of the metal alkali cations separately. Future works using, in addition, other Pt orientations will be performed in order to analyze the effects on the kinetics of these reactions with further information.

## 4 Conclusions

The results presented in this work suggest a dependence of the interfacial water structure on Pt(111) with the nature of the alkali metal cation in the supporting electrolyte as revealed by cyclic voltammetry and pme determination by LITJ experiments. In a hierarchical framework, the electric field determines the water behavior, which is modulated by the ions present in the solution. This effect on the water molecules at the electrode surface by the metal alkali cation, in turn, affects the reactivity, as shown for the inhibition behavior at low potentials of the HPRR. Consequently, the present work paves the way for new future investigations about the influence of the interfacial water structure on the activity of electrocatalytic reactions of interest.

## Supporting Information Available

Table S1 with different constants for Pt and water; Figure S1 with blank voltammograms in the RHE scale, Figures S2, S3, and S4 with the transient results for LiF, NaF, and CsF, respectively, Figure S5 with Δ*E* vs *t*^–1/2^ plots, Figures S6, S7, and S8 with normalized HPRR/HPOR data in RHE scale and non-normalized HPRR/HPOR data in both RHE and SHE scale (PDF)
