# Supplementary material for: Cation Effects on Interfacial Water Structure and Hydrogen Peroxide Reduction on Pt(111)
Source: ACS Meas Sci Au. 2021 Jul 7;1(2):48–55. doi: 10.1021/acsmeasuresciau.1c00004 (PMC9836069; doi:10.1021/acsmeasuresciau.1c00004)
Supplement: Supplementary file 1 — tg1c00004_si_001.pdf [file tg1c00004_si_001.pdf]

SUPPORTING INFORMATION

Cation effects on interfacial water structure and hydrogen peroxide reduction on Pt(111)

Valentín Briega-Martos[†,‡], *Francisco J. Sarabia*[†], *Víctor Climent*[†], *Enrique Herrero*[†],

Juan M. Feliu[†,*]

Instituto de Electroquímica, Universidad de Alicante, Apdo. 99, E-03080, Alicante, Spain

AUTHOR INFORMATION

Corresponding Author

*Juan M. Feliu. Instituto de Electroquímica, Universidad de Alicante, Apdo. 99, E-03080, Alicante, Spain. Email: juan.feliu@ua.es

	Platinum (Pt)	Water
Density / $\boldsymbol{\rho}$ (g/cm^3)	21.5	1
Thermal conductivity /$\boldsymbol{\kappa}$(Jcm^{-1}K^{-1} s^{-1})	0.716	0.006
Heat capacity / **c** (Jg^{-1}K^{-1})	0.133	4.19
Reflectivity at 532nm / **R**	0.636	----

Table S1. Different constants for Pt and water for obtaining the value of ΔT_0.[1]

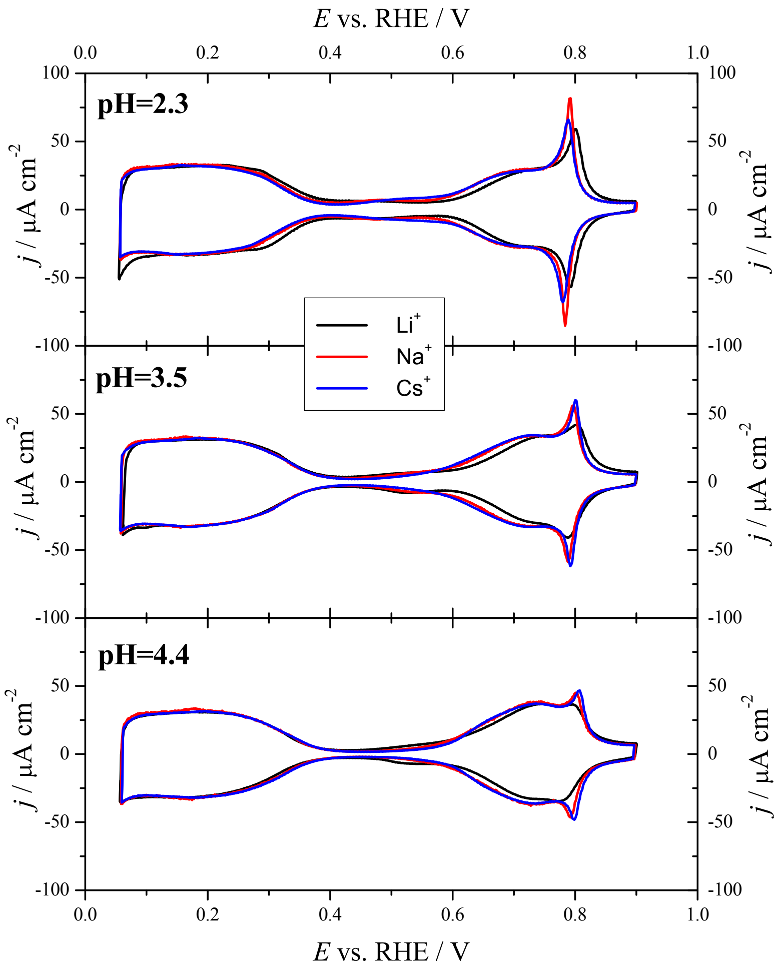

Figure S1. Cyclic voltammograms for Pt(111) in Ar-saturated MeF/$HClO_4$ mixtures (Me^+ = Li^+ (black), Na^+ (red), Cs^+ (blue)) for pH 2.3 (top), 3.5 (center) and 4.4 (bottom) in the RHE scale. Scan rate: 50 mV s^{-1}.

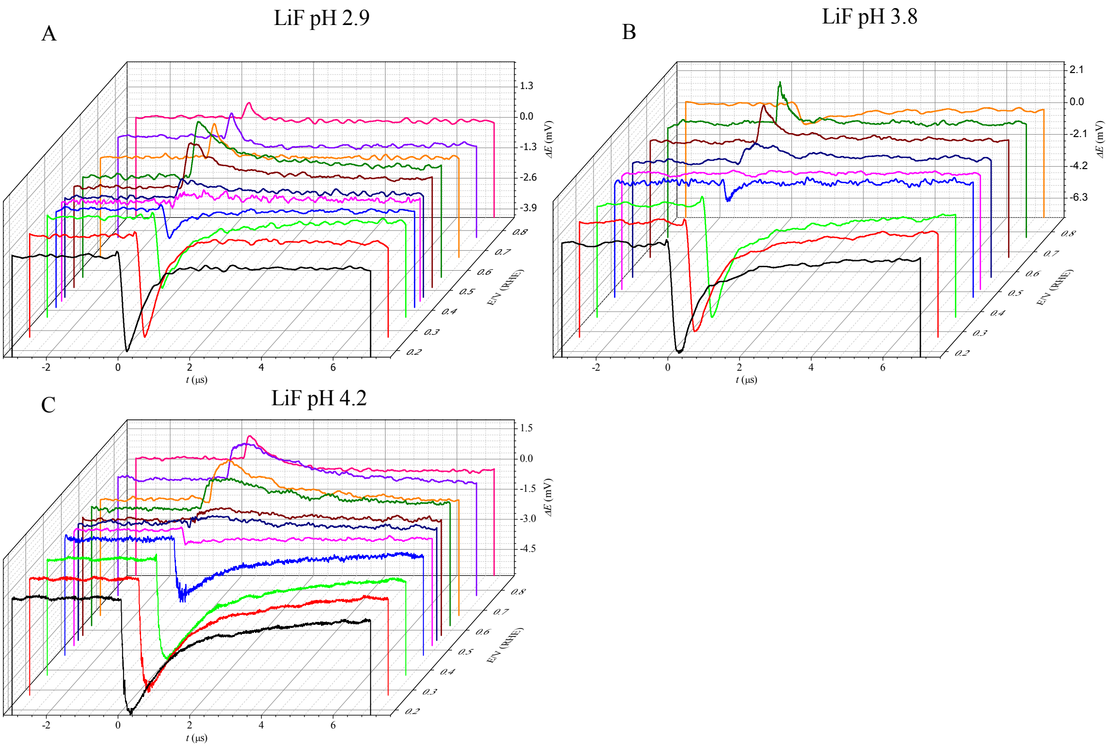

Figure S2. ΔE vs t laser transients recorded for Pt(111) in LiF/$HClO_4$ mixtures at pHs 2.9 (A), 3.8 (B), and 4.2 (C). Laser beam energy: 2 mJ.

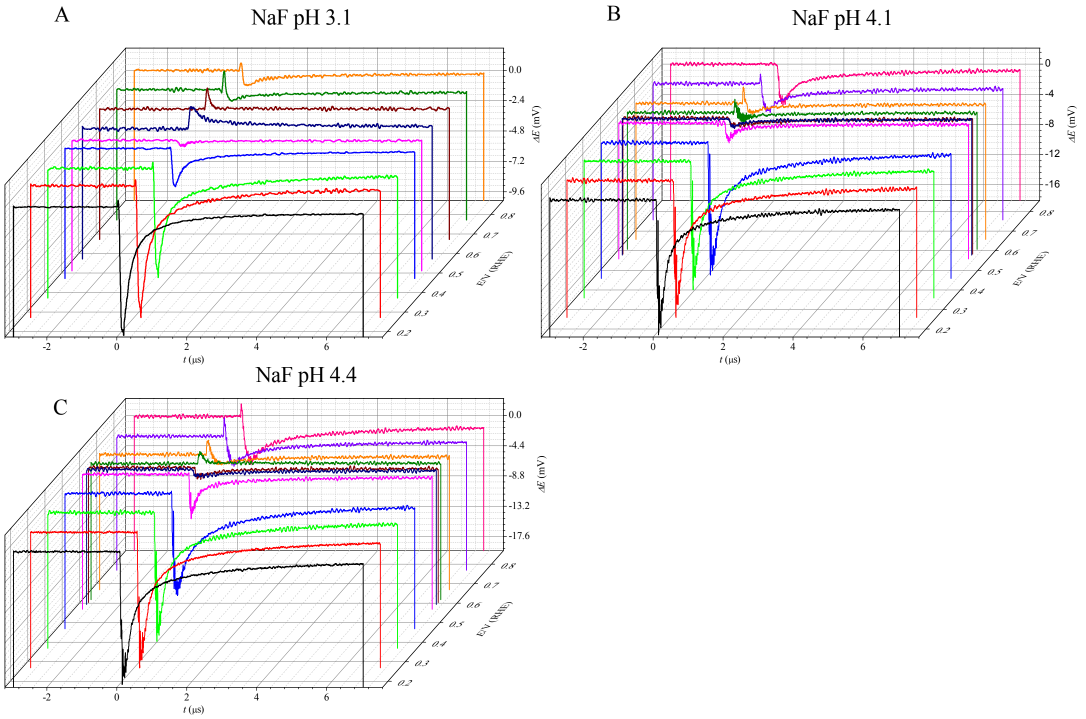

Figure S3. ΔE vs t laser transients recorded for Pt(111) in NaF/$HClO_4$ mixtures at pHs 3.1 (A), 4.1 (B) and 4.4 (C). Laser beam energy: 2 mJ.

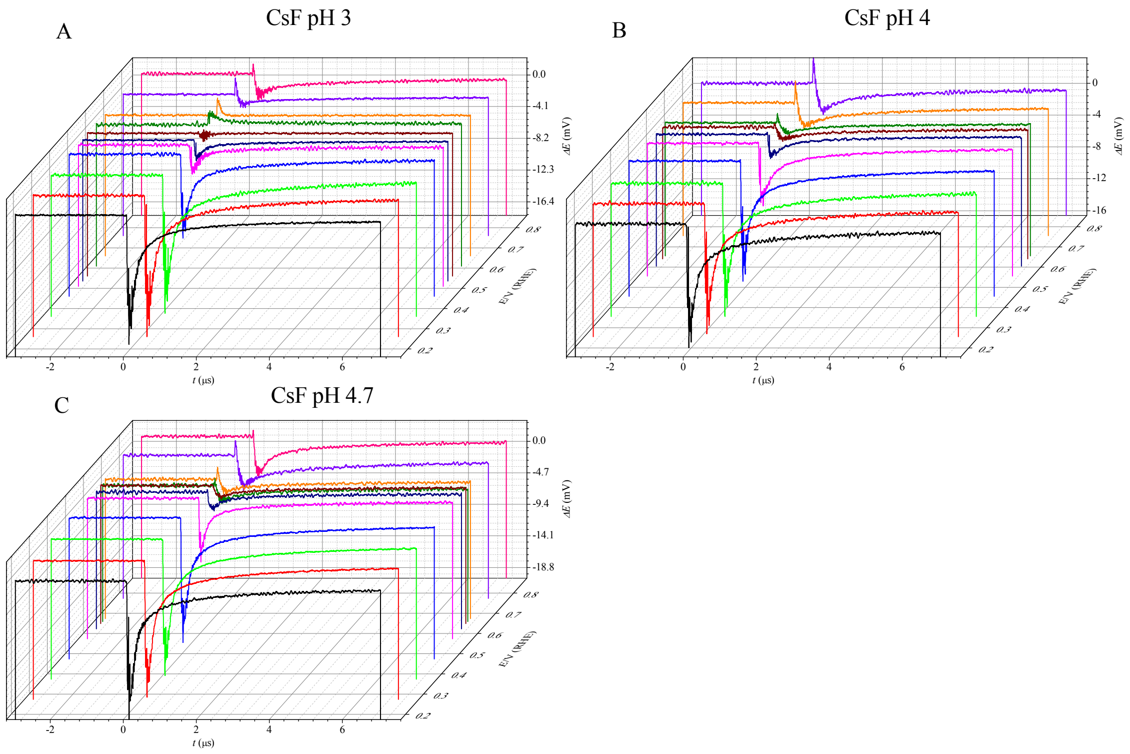

Figure S4. ΔE vs t laser transients recorded for Pt(111) in CsF/$HClO_4$ mixtures at pHs 3 (A), 4 (B) and 4.7 (C). Laser beam energy: 2 mJ.

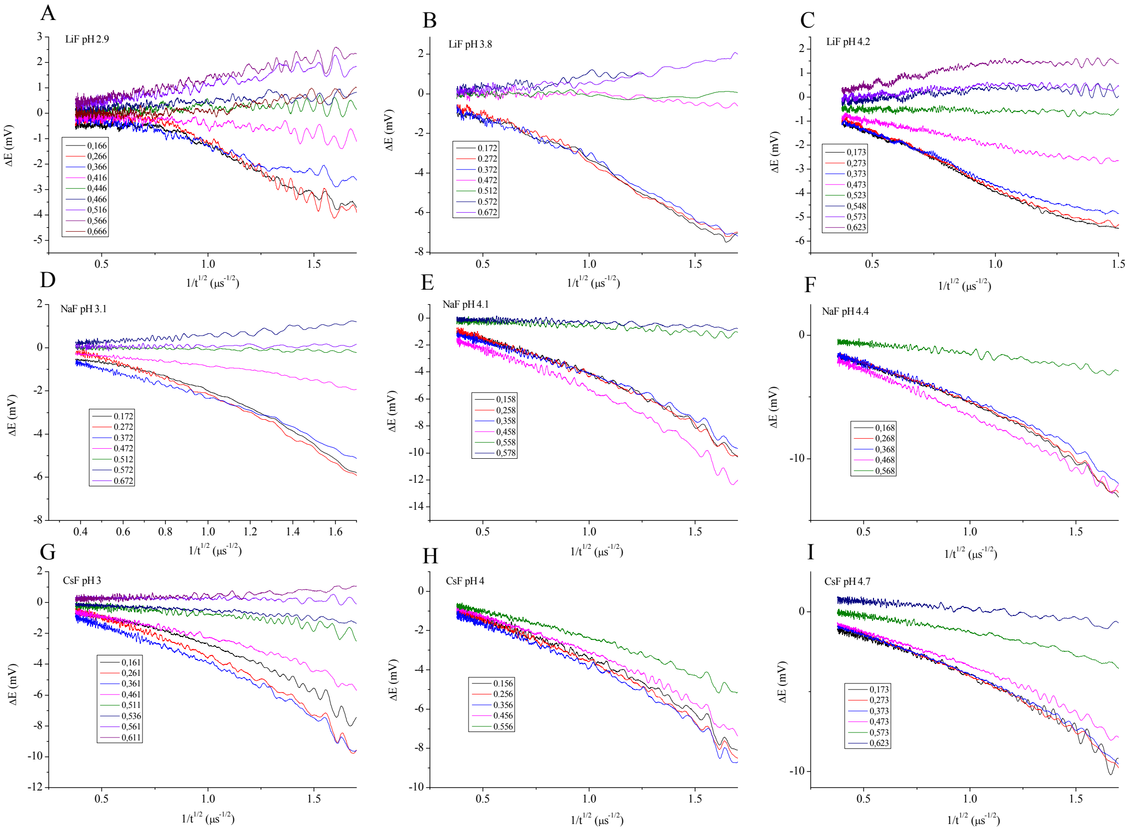

Figure S5. Plot of ΔE vs $1/\sqrt{t}$ at different potentials from which slopes the thermal coefficients have been calculated following the equation (4) of the main text. These data have been obtained from the potential transients recorded for each laser-induced temperature jump experiment in different electrolytes: LiF at pHs 2.9, 3.8 and 4.2 (A, B and C, respectively), NaF at pHs 3.1, 4.1 and 4.4 (D, E and F, respectively), CsF at pHs 3, 4 and 4.7 (G, H and I, respectively).

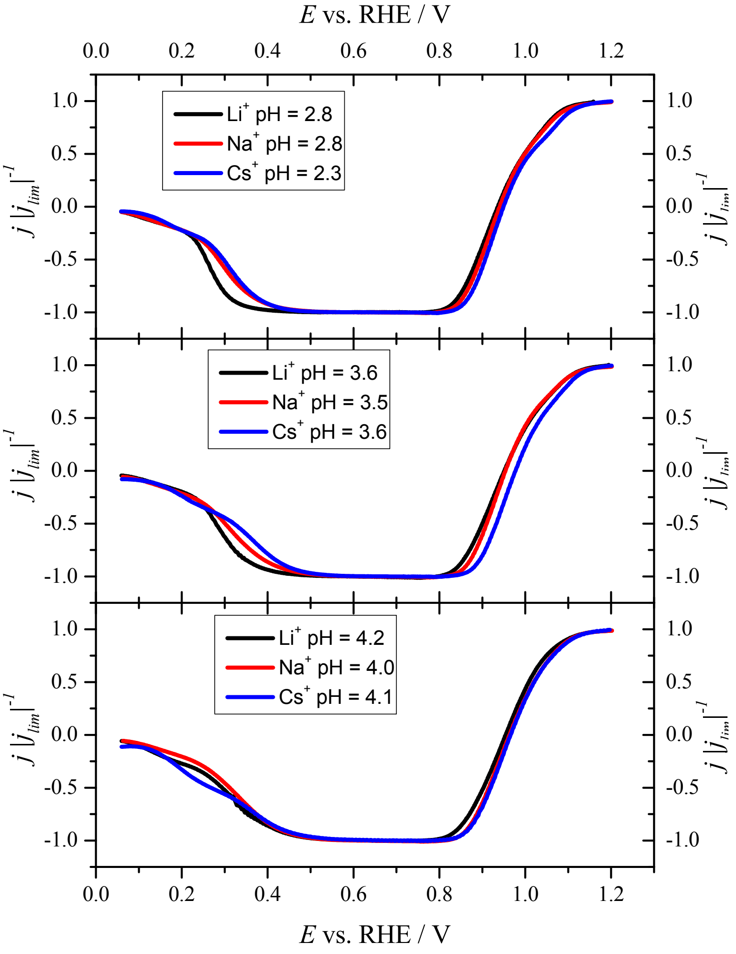

Figure S6. Normalized polarization curves for the HPRR and HPOR on Pt(111) in Ar-saturated MeF/$HClO_4$ mixtures (Me^+ = Li^+ (black), Na^+ (red), Cs^+ (blue)) with 1.7 mM H_2O_2 for different pH values ranging from 2.3 and 4.2 in the RHE scale. Rotation rate: 2500 rpm; scan rate: 50 mV s^{-1}.

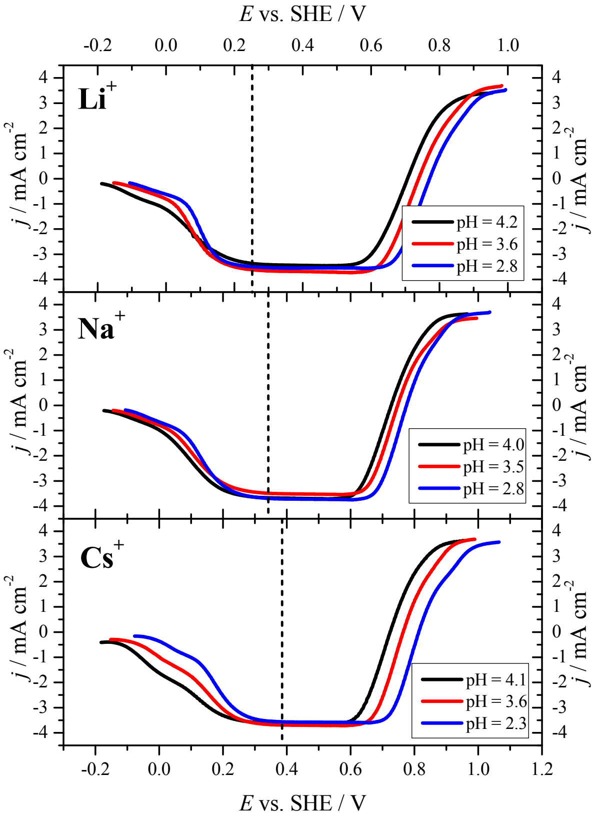

Figure S7. Polarization curves for the HPRR and HPOR on Pt(111) in Ar-saturated MeF/$HClO_4$ mixtures (Me^+ = Li^+ (black), Na^+ (red), Cs^+ (blue)) with 1.7 mM H_2O_2 for different pH values ranging from 2.3 and 4.2 in the SHE scale. Rotation rate: 2500 rpm; scan rate: 50 mV s^{-1}.

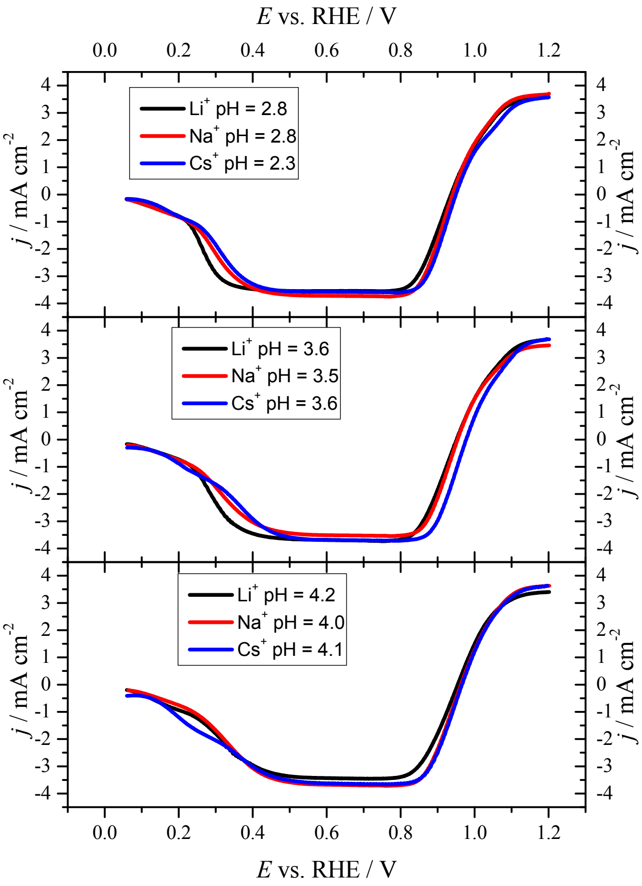

Figure S8. Polarization curves for the HPRR and HPOR on Pt(111) in Ar-saturated MeF/HClO$_4$ mixtures (Me^+ = Li^+ (black), Na^+ (red), Cs^+ (blue)) with 1.7 mM H_2O_2 for different pH values ranging from 2.3 and 4.2 in the RHE scale. Rotation rate: 2500 rpm; scan rate: 50 mV s^{-1}.

AUTHOR INFORMATION

Notes

‡Present address: Forschungszentrum Jülich GmbH, Helmholtz Institute Erlangen-Nürnberg for Renewable Energy (IEK-11), Egerlandstr. 3, 91058 Erlangen, Germany

The authors declare no competing financial interests.

ACKNOWLEDGMENT

Financial support from Ministerio de Ciencia e Innovación (Project PID2019-105653GB-100) and Generalitat Valenciana (Project PROMETEO/2020/063) is acknowledged.

REFERENCES

(1) Weber, M. J. *Handbook of Optical Materials*; CRC Press, 2003.
